# Iowa State Dental Society

**Published:** 1875-08

**Authors:** 


### Proceedings of Societies.

### IOWA STATE DENTAL SOCIETY.

The thirteenth annual meeting of the Iowa State Dental Society, convened in Cedar Rapids, at the Avenue Club Room, Merchant's Block, May 11th, 1875.

Dr. J. T. Abbott, of Manchester, Vice-President of the Society, called the meeting to order, and Dr. R. L. Cochran, of Burlington, was chosen temporary Secretary.

On motion of Dr. P. T. Smith, of Burlington, a committee of three was appointed to examine new applicants for membership. The committee consisted of Drs. P. T. Smith, of Burlington, C. P. Artman, of Waterloo, and J. Hardman, of Muscatine.

The following were in attendance.

Dr. J. T. Abbott, Manchester.
" P. T. Smith, Burlington.
" J. Hardman. Muscatine.
" W. H. Walker, West Branch.
" W. H. Thrift, Eldora.
" E. R. Mullet, Clinton.
" T. A. Hallett, Des Moines.
" G. W. Fuller, Des Moines.
" C. P. Artman, Waterloo.
" T. D. Sturdevant, Clarence.
" G. S. Shattock, Belle Plaine.
" L. C. Ingersol, Keokuk.
" John. M. Booth, Marion.
" A. R. Begun, Cedar Falls.
" Gust North, Springville.
" L. J. Walter, Cedar Rapids.
" J. L. Nott, Marion.

" A. B. Dennis, Muscatine.
" J. L. James, Fairfield.
" A. O. Hunt, McGregor.
" James Thompson, Sigourney.
" C. H. Sterneman, Cedar Rapids.

The Society then adjourned until 2 o'clock p. m.

AFTERNOON SESSION.

The meeting assembled at three o'clock, and President Abbott annouced that the President had arrived and Drs. Wilson and Hanks were appointed to conduct Dr. R. S. Rathbone, the President of the Society, to the chair.

Dr. Rathbone, on taking the chair, made a few appropriate remaks, and said they were ready for buisness.

Dr. Tulloss made a verbal report on the subject of Dental Education.

Dr. Ingersoll gave an interesting statement in regard to this subject in Michigan, and also stated what had been done in Iowa. The regents of the University were in favor of a Dental Department in the University, and he thought that something might be accomplished if the Legislature could be controled.

Dr. Hardman favored such an arrangement as should fully qualify the young men of the state for the practice of the profession of dentistry, and preferred to stay out in the cold to accepting a single chair in the University.

Dr. Abbott favored the position of Dr. Hardman. He thought it better to work and wait until the whole loaf could be secured. He thought it more difficult to get increased appropriations in the Legislature, than to secure an adequate appropriation by proper work in advance of legislative action.

Dr. Ingersol favored a small appropriation to establish a chair of Dentistry even before a Dental Department could be secured.

The further discussion was postponed until to-morrow.

The following new members were then admitted.

Dr. J. M. Booth, Marion.
" O. O. Hunt, McGregor

Dr. H. W. Blockly, Centerville.
" A. D. Dennis, Muscatine.
" E. D. Andrews, Ames.

The following were elected junior members.

Dr. J. H. Nott, Marion.
" T. L. James, Fairfield.
" J. Thompson, Sigourney.

Burlington was designated as the next place of meeting.

On motion of Dr. Ingersoll moved to adjourn until 7;30 this evening.

NIGHT SESSION, 8 O'CLOCK.

Dr. Hardman in the Chair.

The Committee on Membership reported the following, who were duly elected members:

Dr. A. R. Begun, Cedar Falls.
" S. L. Young, Dubuque.
" W. H. Walker, West Branch, junior member.
H. D. Dodge, Dubuque.

Dr. Artman introduced the subject of celluloid for plates.

Dr. Hanks wished to inquire if there was any way of mending the plate when broken.

Dr. Cochran explained a process by which he had succeeded in mending the plate perfectly.

Dr. Wilson thought the camphor taste remained, and only disappeared by not being noticed.

Dr. Booth had used celluloid two years with good success.

Dr. Eaton related a case where the plate had become loose after wearing for a few days but was not able to explain the cause.

Dr. Andruss had mended a plate which had remained for six months, and had not been returned to him.

Dr. Ingersoll never uses a file which produces a surface too smooth. He strives to keep the appearance true to nature. He carves the gum with a knife by which he is able to make it true to nature. His plates have not come back to him, and could not answer the question whether they became discolored. If it does discolor it destroys the charm of its use.

Dr. A. Begun gave his method of using the scraps of celluloid base. He had used them in making full plates, with satisfactory results. He used the steam apparatus.

Dr. Artman gave an amusing experience in the use of celluloid and thought it must have very close attention.

Dr. Smith thought the porosity was caused by over heating. He did not think it caused by a want of pressure ; but rather by the escape of gasses generated by too high heat.

Dr. Morgan said that with the use of steam he had no porous plates. They became porous because of excessive heat. He regarded it a better material than rubber.

On motion of Dr. Shattuck, adjourned to 8:30 o'clock tomorrow morning.

### 2D. DAY, AFTERNOON SESSION, ELECTION OF OFFICERS.

The Society then proceeded to the election of officers for the ensuing year, with the following result.

President—C. P. Artman, Waterloo.

Vice-President—P. T. Smith, Burlington.

Secretary and Treasurer—R. L. Cochran, Burlington.

Corresponding Secretary—I. P. Wilson, Burlington.

Dr. Hardman then called up his Resolution on chloroform and read several extracts, showing the action of other agents condemning the use of chloroform as an anæsthetic agent.

Dr. Walter had used chloroform since its first introduction but had determined to discountenance it.

Dr. Lyon, of Cedar Rapids, gave an interesting case of the use of chloroform and gave it as his opinion that it was giving way to the use of ether. He had been forced to the use of chloroform where ether had failed.

Dr. North, of Cedar Rapids, was also a friend of ether. He had assisted in administering ether and kept the patient under the influence for several hours.

Dr. Hardman thought a small amount of chloroform would kill. If the patient would endure it for a short time he would not regard it dangerous to continue.

Dr. Rigby thought the use of chloroform dangerous and would discourage its use among ignorant practitioners.

Dr. Tulloss was opposed to the resolution and thought we should use everything we can to relieve pain. He considered ether the safer remedy.

Dr. Cochran, of Burlington; Dr. Artman, of Waterloo; Dr, Morgan, of Davenport; Dr. Thrift, of Eldora; and Dr. Abbott, of Manchester, continued the discussion at some length.

Dr. Rigby offered the following as a substitute for Dr. Hardman's resolution:

*Resolved*—That we, as a Society, condemn the use of chloroform as an anæsthetic agent in all dental operations, and that we refrain from using it so far as possible in our practice.

The discussion was further continued by Drs. Cochran, Fuller, Walter, Sternemen, Tulloss and Rigby.

The substitute was then adopted.

Dr. E. R. Mullett presented an essay entitled Clippings, by Dr. E. J. Mullett, of Clinton.

This was a suggestive essay on various topics of practical dentistry. Among these were the following: Taking impressions for partial upper sets; vacancies between the teeth and plate; the use of carbonized paper for articulating a set of teeth; capping nerves, etc. The copy of this paper is so badly interlined that we are unable to present it entire. It contained many valuable suggestions and we regret our inability to place it before our readers.

### CAPPING EXPOSED NERVES.

Dr. Cochran, of Burlington, gave his method of treating exposed nerves and read a number of letters from eminent practitioners both in opposition to and in accord with his method of treatment.

Dr. Morgan agreed in the main with Dr. Cochran, though he used no chloroform to quiet the pain. Acetate of morphia and creosote were used instead. He never killed the nerve.

Dr. Ingersoll related a case of a diseased tooth, removed from his own mouth, filled and returned.

Dr. Fuller also narrated the case of a transplanted tooth which was successful.

Meeting adjourned until 2 o'clock p. m.

AFTERNOON SESSION.

Pres. Rathbun in the chair; minutes read and approved.

Dental Students. Whom shall we take and whom refuse.

Dr. Thrift of Eldora, thought the classical culture of the applicant should be considered, and also their natural adaptability to the work.

Dr. Tulloss, would have the student thoroughly prepared by previous education. The dental profession is advancing.

Dr. Abbott was called to the chair, and the President proceeded to address the Society. Higher qualifications are required to-day than was the case a few years ago. We must eradicate the idea that young men can learn dentistry in a few months of practice, They must be educated and thus we may hope to elevate the standing of the dental profession.

Dr. North coincided with the views expressed by President Rathbun.

Dr. Abbott, of Manchester, said we should examine the natural ability of the applicant, and he would also insist on full scientific attainments. We should lend our influence to encourage the study of physiology and anatomy in our schools. He thought some regulations should be adopted to secure more thorough preparation on the part of students of dentistry.

MISCELLANEOUS.

Dr. Hunt, of McGregor, spoke at some length on the filling of so called ulcerated teeth.

This subject was further discussed by Dr. Shattuck, of Belle Plain, and Dr. Morgan, of Davenport.

Dr. Abbott narrated several cases of office practice, full of amusement and interest.

REVISED CONSTITUTION.

Dr. Wilson, from the committee to revise the constitution of the society made a report, which was accepted.

On motion of Dr. Abbott, the constitution was taken up article by article for approval.

On motion, the constitution, by-laws and order of business were approved as a whole.

On motion of Dr. Hunt, the adoption of the constitution etc., be made the special order of business to follow the roll call of the next meeting.

The President then announced the following standing committees:

Executive committee—Drs. Ingersoll, Morgan and Kulp.

Examining committee—Drs. Abbott, Shattuck and Walter.

Publishing committee—Drs. Dickenson, Hanks and Fuller.

The Bill of the chairman of the executive committee was presented and approved.

On motion of Dr. Wilson, Dr. Walter was authorized to draw on the treaurser for the amount necessary to defray the expenses of printing in the Cedar Rapids Republican.

Proceedings read and approved.

Adjourned.

